# Preliminary Phytochemical Screening, Quantitative Analysis of Alkaloids, and Antioxidant Activity of Crude Plant Extracts from* Ephedra intermedia* Indigenous to Balochistan

**DOI:** 10.1155/2017/5873648

**Published:** 2017-03-13

**Authors:** Rahman Gul, Syed Umer Jan, Syed Faridullah, Samiullah Sherani, Nusrat Jahan

**Affiliations:** ^1^Faculty of Pharmacy, University of Balochistan, Quetta, Pakistan; ^2^Department of Health, Government of Balochistan, Quetta, Pakistan; ^3^Balochistan University of Information Technology, Engineering & Management Sciences (BUITEMS), Quetta, Pakistan; ^4^College of Pharmacy, University of Texas at Austin, Austin, TX, USA

## Abstract

The aim of this study was to evaluate the antioxidant activity, screening the phytogenic chemical compounds, and to assess the alkaloids present in the* E. intermedia* to prove its uses in Pakistani folk medicines for the treatment of asthma and bronchitis. Antioxidant activity was analyzed by using 2,2-diphenyl-1-picryl-hydrazyl-hydrate assay. Standard methods were used for the identification of cardiac glycosides, phenolic compounds, flavonoids, anthraquinones, and alkaloids. High performance liquid chromatography (HPLC) was used for quantitative purpose of ephedrine alkaloids in* E. intermedia*. The quantitative separation was confirmed on Shimadzu 10AVP column (Shampack) of internal diameter (id) 3.0 mm and 50 mm in length. The extract of the solute in flow rate of 1 ml/min at the wavelength 210 nm and methanolic extract showed the antioxidant activity and powerful oxygen free radicals scavenging activities and the IC50 for the* E. intermedia* plant was near to the reference standard ascorbic acid. The HPLC method was useful for the quantitative purpose of ephedrine (E) and pseudoephedrine (PE) used for 45 samples of one species collected from central habitat in three districts (Ziarat, Shairani, and Kalat) of Balochistan. Results showed that average alkaloid substance in* E. intermedia* was as follows: PE (0.209%, 0.238%, and 0.22%) and E (0.0538%, 0.0666%, and 0.0514%).

## 1. Introduction

The importance of medicinal plant in drug development is known to us and humans have used them for different diseases from the beginning of human history [1]. Traditional folk treatment from wild plants has always guided researchers to search for novel medications to develop healthy life for humans and animals [2]. In addition, some medicinal plants are still obscured within the plant which need to be scientifically evaluated.

**Figure 1:**
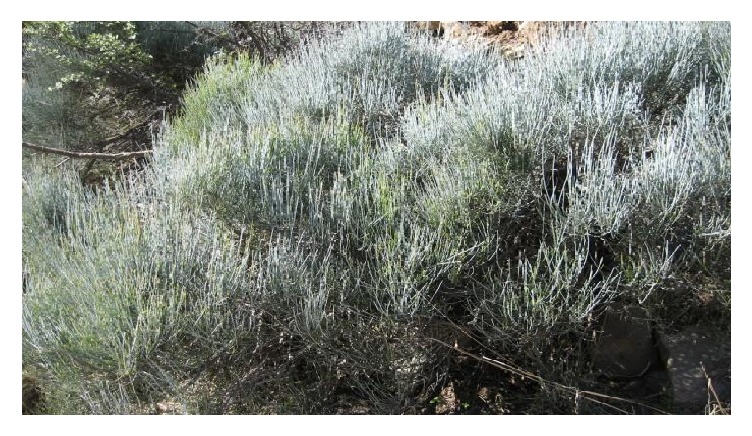
*Ephedra intermedia* plant.

*Ephedra (Ephedra intermedia) *belongs to family Ephedraceae and is a genus of nonflowering plants, related to Gnetales, very near relatives of angiosperms [3]. Majority of the 50* Ephedra* species throughout the world are adapted as a shrub to moisture and desert conditions [4–6]. Three species are found in Pakistan.* E. intermedia* shrubs are always green called Ma-Huang and, locally in Balochistan, they are called Oman.* Ma-Huang (Ephedra)* is resultant from the aerial parts of* Ephedra sinica* Stapf,* E. intermedia* Stapf,* E. equisetina* Bunge, and* E. distachya* L. It has been utilized medicinally as a stimulant, diaphoretic, and antiasthmatic [7, 8]. It is a xerophytic shrub plant and grows in unfavorable soil and climatic conditions such as high temperature and high light (Figure 1) [9].

Most of the marketed drugs of* Ephedra* extracts are taken from the ephedrine and pseudoephedrine alkaloids present in many species shoots. The best recognized drug prepared from* Ephedra* is Ma-Huang utilized in Chinese drugs for the treatment of nasal congestion, fever, and asthma [10]. Ma-Huang is also used as a respiratory sedative and cough treatment. Herbal mixture containing Ma-huang and combination products are widely available in health food stores. Many of these products are marketed as “diet pills” or “energy pills” or both [11]. Ma-Huang was traditionally gained from dried stem of* E. equisetina, E. sinica,* and* E. intermedia* [12] found in the region of Iran, Northwest India, and Pakistan (Balochistan). These shrub plants also showed antioxidant and antimicrobial activities [13–15].* Ephedra* basic compounds consist of the alkaloids ephedrine and pseudoephedrine and phenols [16]. The stem consists of overall 1–3% alkaloids, having ephedrine comprising 30–70% of the total, depending on all the species and types of* Ephedra* plant. Ephedrine activates the CNS, increases the blood pressure, dilates the bronchial tubes, and increases the pulse rate. Pseudoephedrine is used for the relief of nasal congestion in its synthetic form [17–19]. HPLC method for the quantitative analysis [20] can give a baseline resolution of the alkaloids with the advantage of simple extraction and direct analysis of the alkaloids without derivatives: the reversed-phase HPLC method [21].

Balochistan is the largest and driest province of the country (about 35,000 sq km, i.e., 44% of the country's total area). It lies north to the tropics, between the latitude of 24 and 32 and between the longitudes of 60 to 70 East, with an area of about 134,000 square miles and a population of 140 million [22]. Thus, main purpose of this research work is to analyze the phytochemical screening and quantitative estimation of alkaloids and antioxidant activity of crude* Ephedra *extract.

## 2. Material and Methods

Aerial parts of* Ephedra* were collected in June to September 2015-2016 in five different places of Balochistan. All plants were growing at an altitude ranging from 600 to 1100 m over the sea level. These plants were identified at the herbarium section; a voucher specimen (E-RBT-04) has been deposited in the Department of Botany, University of Balochistan, Quetta, Pakistan.

Hydroalcohlic mixture was prepared by mixing two liters each of analytical grade ethanol, methanol and distilled water 7 : 3. the plant* Ephedra* was collected and cut into thin slices by minicing appratus. 2 kg of material was weighted and put into the brown glass bottle. Hydroalcholic mixture was added to it and macerated for one weak. The bottle was sealed with aluminium foil and kept in labortory at room temperature, and the bottle was shaken after 24 hours. Finally the filtrate was filtered through many layers of muslin cloth for coarse filtration. The coarse filtrate was than filtered through Whatman number 1 filter paper. The obtained filtrate was evaporated in a vacuum rotary evaporator under reduced pressure at 40°C until the filtrate was reduced to one-third of the starting filtrate volume. The obtained extracts were collected in stopper glass bottles and stored at 0°C.

## 3. Quantitative Analysis of the Alkaloids by HPLC

A simple and rapid HPLC method was used for determination of alkaloids in different samples of* Ephedra* species by comparing it with the standards of ephedrine and pseudoephedrine. HPLC is valid method for the identification of ephedrine (E) and pseudoephedrine (PE) in* Ephedra* raw herbs. The pseudoephedrine and ephedrine calibration curve range in between 0.03125 to 5 microgram per milliliter. The calibration curve of the plant extract was made having regression coefficient of 0.9998 with different retention time. Different ephedrine alkaloids were eluted from the extracted* Ephedra* plant with 0.25 mol/H3PO4 water solution. The HPLC quantitative separation was confirmed on Shimadzu 10AVP column (Shampack) of internal diameter (id) 3.0 mm and 50 mm in length. The extract of the solute was in flow rate of 1 ml/min with mobile phase buffer solution pH 5.3 and methanol and acetonitrile in ratio 1 : 1 : 8. The recognition wavelength was put at 210 nm. Reference standards for (−)-ephedrine HCl, (+)-pseudoephedrine HCl, and ascorbic acid were received from Merck Serono, Quetta Factory, and commercial market. All other reagents and chemicals were used of analytical grade.

### 3.1. Phytochemical Qualitative Analysis

The plant extracts and methanolic and ethanolic aqueous solutions were assessed for the existence of the phytochemical analysis by using the following standard methods [23–26].

#### 3.1.1. Test for Anthraquinones

10 ml of benzene was added in 6 g of the* Ephedra* powder sample in a conical flask and soaked for 10 minutes and then filtered. Further 10 ml of 10% ammonia solution was added to the filtrate and shaken vigorously for 30 seconds and pink, violet, or red color indicated the presence of anthraquinones in the ammonia phase.

#### 3.1.2. Test for Tannins

10 ml of bromine water was added to the 0.5 g aqueous extract. Decoloration of bromine water showed the presence of tannins.

#### 3.1.3. Test for Saponins

5.0 ml of distilled water was mixed with aqueous crude plant extract in a test tube and it was mixed vigorously. The frothing was mixed with few drops of olive oil and mixed vigorously and the foam appearance showed the presence of saponins.

#### 3.1.4. Tests for Flavonoids

*Shinoda Test*. Pieces of magnesium ribbon and Hcl concentrated were mixed with aqueous crude plant extarct after few minutes and pink color showed the presence of flavonoid.

*Alkaline Reagent Test*. 2 ml of 2.0% NaOH mixture was mixed with aqueous plant crude extract; concentrated yellow color was produced, which became colorless when we added 2 drops of diluted acid to mixture. This result showed the presence of flavonoids.

#### 3.1.5. Tests for Glycosides

*Liebermann's Test*. We added 2.0 ml of acetic acid and 2 ml of chloroform with whole aqueous plant crude extract. The mixture was then cooled and we added H_2_SO_4_ concentrated. Green color showed the entity of aglycone, steroidal part of glycosides.

*Keller-Kiliani Test*. A solution of glacial acetic acid (4.0 ml) with 1 drop of 2.0% FeCl_3_ mixture was mixed with the 10 ml aqueous plant extract and 1 ml H_2_SO_4_ concentrated. A brown ring formed between the layers which showed the entity of cardiac steroidal glycosides.

*Salkowski's Test*. We added 2 ml H_2_SO_4_ concentrated to the whole aqueous plant crude extract. A reddish brown color formed which indicated the presence of steroidal aglycone part of the glycoside.

#### 3.1.6. Test for Terpenoids

2.0 ml of chloroform was added with the 5 ml aqueous plant extract and evaporated on the water path and then boiled with 3 ml of H_2_SO_4_ concentrated. A grey color formed which showed the entity of terpenoids.

#### 3.1.7. Test for Steroids

2 ml of chloroform and concentrated H_2_SO_4_ were added with the 5 ml aqueous plant crude extract. In the lower chloroform layer red color appeared that indicated the presence of steroids.

### 3.2. Antioxidant Activity

Method used for antioxidant activity was DPPH free radical scavenging assay.

#### 3.2.1. DPPH Free Radical Scavenging Assay

To determine antioxidant activity 2,2-diphenyl-1-picryl-hydrezyl (DPPH) was used as free radical. 100 *µ*M concentration of DPPH was used in methanol. Serial dilutions were made to check the IC50. In 96-well micro plate total volume was 100 *µ*l which was consisting of 90 *µ*l of DPPH solution and 10 *µ*l of the test solution. The contents were mixed and incubated for 30 minutes at 37°C. To determine the absorbance at 517 nm synergy HT BioTek USA micro plate reader was used. Ascorbic acid was used as standard antioxidant [27]. All readings were taken in triplicate. Ez-fit-5, Perrella Scientific Inc., Amherst, USA, software was used to calculate the IC50. Decrease in absorbance indicated increased radical scavenging activity which was determined by the following formula:

(1) Inhibition%=abs.  of  control−abs.  of  test  solution×100Abs.  of  control,

where absorbance of control = total radical activity without inhibitor and absorbance of test = activity in the presence of test compounds.

## 4. Results and Discussion

The HPLC analysis explained here was shown to be a correct and accurate technique for the illustration of E and PE in* E. intermedia*. Although liquid chromatographic techniques for quantitating ephedrine alkaloids in simple* Ephedra* herb have been explained previously [28].

**Figure 2:**
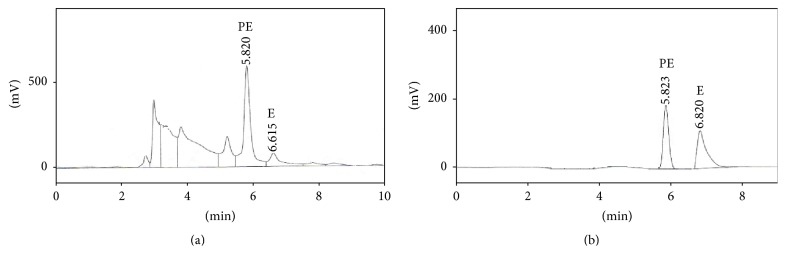
(a) Representative chromatograms of extracts from several* E. intermedia* containing (E) ephedrine and (PSE) pseudoephedrine. (b) Reference standard.

A distinctive HPLC chromatogram for standard solution of E and PE is shown in Figure 2. Retention times examined for PE and E were between about 5.82 and 6.82 minutes. Relative retention times for each alkaloid were 5.7 and 6.61 minutes.

When extracted independently, no chromatographic interference was observed for any of the extra ingredients explained in Table 1.

The optimized technique was performed to the HPLC analysis of 45* E. intermedia* samples.* Ephedra* herb was collected in three regions of Ziarat, one region of Sherani, and two regions of Kalat. The information on E and PE content in these samples is discussed in Table 1.

The outcome or result demonstrated that the whole quantity of these two alkaloids were not considerably different among the species, but the ratio pattern of the alkaloid content was established to be helpful in classifying the samples resulting from this* Ephedra* species. The average recovery of pseudoephedrine is as follows: Ziarat 0.2186%, Sherani 0.238%, and Kalat 0.2255% and in the same solution the recovery of Ephedrine in Ziarat was 0.0538%, in Sherani was 0.0666%, and in Kalat was 0.0514% and the relative standard deviation for each active substance was also calculated. The result was summarized in Table 2.

The results in tables show excellent recovery of PE from its solution and very low adsorption of E. High recoveries of PE were obtained due to its higher polarity and solubility that provided a strong interaction with orthophosphoric acid allowing it to remain in the aqueous solution. However, the loss of E might be caused due to its lower polarity and less quantity in* Ephedra *Herba.

The consequences illustrated that the whole quantity of these two alkaloids did not considerably vary among the samples. But the ratio pattern of the alkaloids content was originated to be helpful in recognizing the samples resulting from these* Ephedra* species and the ratio pattern was extra precise compared to that accounted before [29]. The results of this study confirm the results of a previous study [30]. The ratio E/total alkaloids was created to be extremely helpful in totally recognizing* E. intermedia* (ratio < 0.31, for all samples). Hong et al. [30] stated the ratio for* E. intermedia* was < 3.2. We employed this rule to recognize the species of* Ephedra* using the alkaloid content data accounted by other scientists [28, 29]. The results illustrated that more than 95% of the* Ephedra* herb samples resulting from the one* Ephedra* plant could be recognized accurately.

### 4.1. Phytochemical Analysis for the Methanolic and Ethanolic Extract of* Ephedra intermedia*

The detailed methods are described above and the results are also given in Table 3.

### 4.2. Antioxidant Activity Using Ascorbic Acid as Standard Equivalent

**Figure 3:**
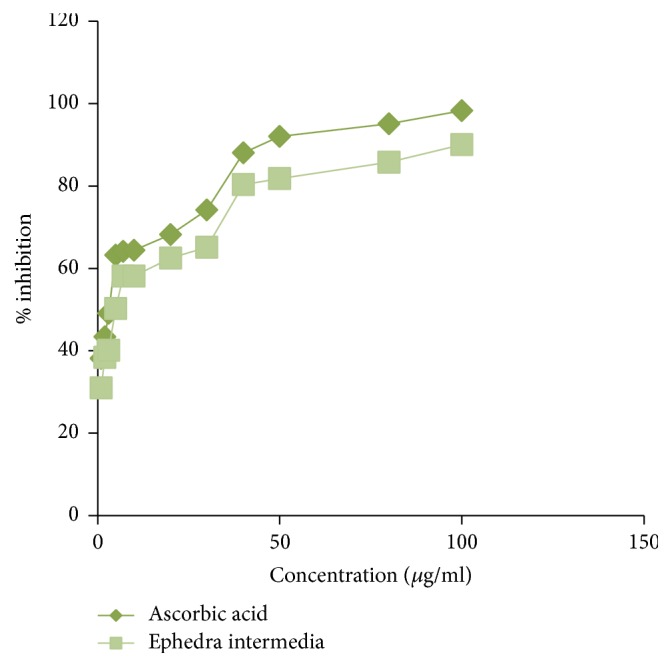
% inhibition of ascorbic acid standard and* Ephedra intermedia* extract.

The methanolic extract free radical scavenging activity of* Ephedra intermedia* has been tested by DPPH radical procedure using ascorbic acid as a reference standard. The concentration ranged from 1 to 100 *µ*g/ml. The zero inhibition was measured for the solution which contained only DPPH without any aqueous plant extract. The results are showed in Table 4 and the data readings are explained in Figure 3.

## 5. Conclusion

The phytochemical analysis showed that the* Ephedra intermedia* plant extract contains a mixture of phytochemicals as reducing sugars, cardiac glycoside, phenolic compounds, flavonoids, and alkaloids. The quantitative DPPH assay indicated that the plant extract has potent antioxidant activity which can be an excellent option for biological and chemical analysis and can be further subjected for the isolation of the therapeutically active compounds and the alkaloid content of PE in* E. intermedia* of Shairani (average 1.524 mg/500 mg) was higher than that in* E. intermedia* of Ziarat (average 1.36 mg/500 mg) and* E. intermedia* of Kalat (average 1.35 mg/500 mg), but the changeable range of total alkaloid content of each* Ephedra* was so broad that the whole alkaloid content ranges of these collected samples species really overlap, which cannot affect the claim that these* Ephedra* species should be analyzed as dissimilar drugs. The contents of PE and E are also pretty different between the samples of* E. intermedia*. In this study we utilized the method for the recognition of* E. intermedia* by means of the ratio E/total alkaloids. Since these samples are not surely different in alkaloid contents of E/PE as well, this method of study is an excellent choice and can be further subjected for the isolation of therapeutically active substance with antiasthmatic and antioxidant potency.

## Figures and Tables

**Table 1:** Contents of two alkaloids in 45 *Ephedra* samples derived from the *E. intermedia in* (500 mg powder).

Collection place	PE	E	Total	PE/total	E/total
Ziarat (Warchum)	1.318	0.017	1.335	0.98727	0.01273
Ziarat (Warchum)	1.026	0.283	1.309	0.7838	0.2162
Ziarat (Warchum)	1.163	0.351	1.514	0.76816	0.23184
Ziarat (Warchum)	1.173	0.334	1.507	0.77837	0.22163
Ziarat (Warchum)	0.645	0.046	0.691	0.93343	0.06657
Ziarat (Warchum)	0.795	0.136	0.931	0.85392	0.14608
Ziarat (Warchum)	1.173	0.305	1.478	0.79364	0.20636
Ziarat (Kawas)	1.307	0.263	1.57	0.83248	0.16752
Ziarat (Kawas)	1.316	0.304	1.62	0.81235	0.18765
Ziarat (Kawas)	1.289	0.32	1.609	0.80112	0.19888
Ziarat (Kawas)	1.433	0.392	1.825	0.78521	0.2148
Ziarat (Kawas)	1.415	0.401	1.811	0.78133	0.2214
Ziarat (Proper)	1.393	0.376	1.769	0.78745	0.21255
Ziarat (Proper)	1.411	0.404	1.815	0.77741	0.22259
Ziarat (Proper)	1.381	0.363	1.744	0.79186	0.20814
Ziarat (Proper)	1.379	0.355	1.734	0.79527	0.20473
Ziarat (Warchum)	0.613	0.183	0.796	0.7701	0.2299
Ziarat (Warchum)	0.546	0.143	0.689	0.79245	0.20755
Ziarat (Warchum)	0.597	0.18	0.777	0.76834	0.23166
Ziarat (Warchum)	1.118	0.049	1.167	0.95801	0.04199
Ziarat (Warchum)	1.119	0.198	1.317	0.84966	0.15034
Ziarat (Kawas)	0.873	0.217	1.09	0.80092	0.19908
Ziarat (Kawas)	1.0996	0.382	1.4816	0.74217	0.25783
Ziarat (Kawas)	1.0992	0.38	1.4792	0.7431	0.2569
Ziarat (proper)	1.099	0.374	1.473	0.7461	0.2539
Ziarat (proper)	0.867	0.28	1.147	0.75589	0.24412
Ziarat (proper)	0.859	0.22	1.079	0.79611	0.20389
Ziarat (proper)	0.843	0.227	1.07	0.78785	0.21215
Ziarat (proper)	1.374	0.332	1.706	0.80539	0.19461
Shairani	1.226	0.262	1.488	0.823	0.176
Shairani	0.873	0.222	1.095	0.797	0.202
Shairani	1.007	0.403	1.41	0.714	0.285
Shairani	0.993	0.399	1.392	0.713	0.286
Shairani	0.996	0.468	1.464	0.68	0.319
Shairani	1.49	0.313	1.803	0.826	0.173
Shairani	1.475	0.307	1.782	0.827	0.172
Shairani	1.466	0.295	1.761	0.832	0.167
Kalat (Harboi)	1.009	0.179	1.188	0.849	0.1506
Kalat (Harboi)	1.374	0.346	1.72	0.798	0.2011
Kalat (Harboi)	1.514	0.32	1.834	0.825	0.1744
Kalat (Harboi)	1.106	0.195	1.301	0.85	0.1498
Kalat (Harboi)	1.188	0.217	1.405	0.845	0.1544
Kalat (Harboi)	0.87	0.274	1.144	0.76	0.2395
Kalat (Harboi)	0.867	0.264	1.131	0.766	0.2334
Kalat (Harboi)	0.872	0.266	1.138	0.766	0.2337

**Table 2:** Recovery and SD of E and PE of studied samples. *n* = 45.

Area	PE			E		
	Mean	SD	Recovery%	Mean	SD	Recovery%
Ziarat, *n* = 29	1.093	0.276	0.2186	0.269	0.1129	0.0538
Shairani, *n* = 8	1.190	0.256	0.238	0.333	0.0821	0.0666
Total, *n* = 45	1.112	0.0155	0.222	0.278	0.0275	0.0204

**Table 3:** Phytochemical analysis for the methanolic and ethanolic extract of *E.intermedia*.

Phytochemical compounds	Methanolic extract	Ethanolic extract
Cardiac glycosides	+	+
Saponin glycoside	−	−
Alkaloids	+	+
Amino acids	−	−
Starch	−	−
Reducing sugars	+	+
Phenols	+	+
Volatile oil	−	−
Tannin	−	−
Flavonoids	+	+
Steroids	−	+

+ = presence; − = absent.

**Table 4:** Percentage inhibition activity for ascorbic acid and *E. intermedia*.

Concentration *µ*g/ml	% inhibition by ascorbic acid ± SD	% inhibition by *Ephedra intermedia * ± SD
1	38.15 ± 1.01	30.94 ± 1.01
2	43.35 ± 1.14	38.38 ± 1.12
3	49.09 ± 1.09	40.09 ± 1.18
5	63.19 ± 1.11	50.29 ± 0.76
7	64.10 ± 1.03	58.16 ± 1.11
10	64.38 ± 1.11	58.16 ± 1.33
20	68.19 ± 2.11	62.46 ± 1.35
30	74.21 ± 1.13	65.17 ± 1.71
40	88.10 ±1.50	80.36 ± 1.22
50	92.01 ± 1.10	81.83 ± 1.49
80	95.12 ± 1.22	85.83 ± 1.01
100	98.33 ± 1.28	90.08 ± 1.37
